# Safety and efficacy of percutaneous Watchman 2.5 device versus Amplatzer Amulet for left atrial appendage closure in patients with non-valvular atrial fibrillation: A systematic review and study-level meta-analysis

**DOI:** 10.1371/journal.pone.0295804

**Published:** 2024-02-14

**Authors:** Farah Yasmin, Eman Ali, Abdul Moeed, Farwa Zaidi, Muhammad Umar, Vikash Virwani

**Affiliations:** 1 Yale University School of Medicine, New Haven, CT, United States of America; 2 Department of Medicine, Dow University of Health Sciences, Karachi, Pakistan; 3 IU Health Ball Memorial Hospital, Muncie, IN, United States of America; 4 Medical College, Aga Khan University, Karachi, Pakistan; BSMMU: Bangabandhu Sheikh Mujib Medical University, BANGLADESH

## Abstract

**Introduction:**

In patients with non-valvular atrial fibrillation (NVAF), mechanical occlusion of the left atrial appendage (LAA) using a permanently implanted device may be an effective alternative to oral anti-coagulants (OAC). To facilitate left atrial appendage closure (LAAC), multiple percutaneous devices have been proposed. Watchman Generation 2.5 and Amplatzer Amulet are the two most popular used devices for preventing stroke in patients with NVAF. We sought to compare safety and efficacy outcomes between Watchman 2.5 and Amplatzer Amulet in patients undergoing LAAC procedure.

**Methods:**

We carried out a comprehensive and systematic search of the databases PubMed and Scopus, for all studies that compared the safety and efficacy of Watchman 2.5 and Amplatzer Amulet devices, from inception, till June 2023. We performed the statistical analysis using Review Manager (V.5.4.1 Cochrane Collaboration, London, United Kingdom). The safety outcomes of interest included device success, device-related thrombus, device embolization perioperatively and at follow-up, perioperative pericardial perfusion events, and perioperative cardiac tamponade events. Efficacy outcomes were all-cause mortality perioperatively and at follow-up, cardiovascular (CV) mortality at follow-up, stroke, major and minor bleeding events at follow-up, transient ischemic attack (TIA) in follow-up period, thromboembolic events in follow-up period, and peri-device leakage in perioperative period. All data was analysed using a random-effects model, and presented as risk ratios (RRs) with 95% confidence intervals (95%CIs).

**Results:**

Regarding safety outcomes, device success was non-significantly reduced in Watchman group when compared with Amulet (RR 0.99, p = 0.57; I^2^ = 34%). In contrast, device-related thrombus was non-significantly increased in Watchman 2.5 group in comparison to Amulet (RR 1.44, p = 0.11; I^2^ = 0%). There was no significant difference between the devices in terms of device embolization in the perioperative (RR 0.36, p = 0.38; I2 = 22%) and follow-up (RR 2.24, p = 0.13; I^2^ = 0%) periods. Likewise, there was no significant difference in the risks of pericardial effusion (RR 0.98, p = 0.98; I^2^ = 0%), and cardiac tamponade (RR 0.65, p = 0.76; I2 = 62%) perioperatively. Regarding efficacy outcomes, no significant difference was observed in all-cause mortality between devices perioperatively (RR 0.51, p = 0.32; I^2^ = 0%) and at follow-up (RR 1.08, p = 0.56; I^2^ = 0%). CV-mortality was non-significantly reduced in Watchman group when compared with Amulet (RR 0.57, p = 0.20; I^2^ = 0%). The Amulet device was not superior to the Watchman device in terms of stroke at follow-up (RR 1.13, p = 0.63; I^2^ = 0%). Sub-group analysis showed comparable ischaemic and haemorrhagic stroke events between two devices. Furthermore, at follow-up, there was no significant difference in major (RR 1.06, p = 0.63; I^2^ = 0%) and minor bleeding events (RR 1.81, p = 0.17; I^2^ = 0%) between the two devices. No difference was observed for trans-ischemic attack (RR 1.89, p = 0.24; I^2^ = 0%) and thromboembolic events (RR 0.96, p = 0.96; I^2^ = 0%) at follow-up. No significant difference was observed between devices for peri-device leakage in perioperative period (RR 2.16, p = 0.05; I^2^ = 0%).

**Conclusion:**

The data suggested that LAAC is safe and efficacious procedure irrespective of device used, with generally low complication rates. Watchman generation 2.5 remains non-superior to Amplatzer Amulet in terms of safety and efficacy outcomes.

## Introduction

Non-valvular atrial fibrillation (NVAF) diagnosed patients have a 3-5-fold higher risk of ischemic stroke due to blood flow stoppage in the left atrial appendage (LAA), which enhances local thrombus formation [1]. Oral anticoagulation (OAC) is considered an effective treatment for prevention of thromboembolic events. Conversely, their use has been restricted as a consequence of poor patient compliance, need for long-term treatment, side effects, drug interactions and contraindications such as bleeding [2]. In addition, left atrial appendage occlusion (LAAO) procedure is non-inferior to direct oral anticoagulants (DOACs) for reducing bleeding, cardio-embolic events, cardiovascular death, or procedure-related complications in patients with NVAF who are at considerable risk for stroke, as demonstrated in *Left Atrial Appendage Closure vs*. *Novel Anticoagulation Agents in Atrial Fibrillation (*PRAGUE-17). In the long-term follow-up of PRAGUE-17, LAAO remained superior to novel oral anticoagulants (NOACs) [3].

In patients with NVAF, mechanical occlusion of the left atrial appendage (LAA) using a permanently implanted device may be an effective alternative to OAC for stroke prevention. To facilitate left atrial appendage closure (LAAC), several percutaneous devices have been proposed. All patients who are contraindicated for long-term OAC are currently advised to get treated with LAAO device implantation in accordance with the European guidelines on atrial fibrillation (class IIb) [4]. Based on favourable outcomes in PROTECT-AF *(Watchman Left Atrial Appendage Closure Technology for Embolic Protection in Patients With Atrial Fibrillation)* and PREVAIL *(Evaluation of the Watchman LAA Closure Device in Patients With Atrial Fibrillation Versus Long Term Warfarin Therapy)*, which were the first randomized controlled trials (RCTs) to compare the clinical efficacy and safety of LAAC with the Watchman device to warfarin therapy in patients with NVAF, the Food and Drug Administration (FDA) approved single-seal mechanism Watchman device for LAAO in 2015 [5, 6]. In December 2008, the AMPLATZER Cardiac Plug (ACP) received CE Mark. It was the first AMPLATZER device that Dr. Kurt Amplatz designed specifically for appendage closure [7]. Based on positive findings of several observational studies and RCTs including Amulet IDE randomized trial, the amulet device was recently authorized by FDA [8].

Despite favourable outcomes, both the Watchman 2.5 and Amplatzer Amulet devices are not devoid of complications. A multi-centre prospective real-world registry reported major serious adverse events rate to be 3.2% among 1088 participants undergoing LAAO with the Amulet device [9]. Similarly, in a study analysing 1021 patients of LAAO with the Watchman device, the rate of serious adverse events was demonstrated to be 2.8% [10]. Several observational studies and RCTs comparing implant success, procedural outcomes, and safety events between two devices have been published till date. However, in terms of device-related complications, contradictory results have been obtained. To further evaluate safety and efficacy of both devices, the ORIGINAL *(saxOnian RegIstry analyzinG and followINg left atrial Appendage cLosures)* registry, an open-label multicentre, prospective clinical registry study was recently conducted which supported thesis from previous large, randomized trials that LAAC can be performed with a very high procedural success rate in the everyday clinical routine irrespective of the used LAA device (Watchman 2.5 generation or Amplatzer Amulet) [11]. Given the limitations of existing data and potential impact of these two devices in the AF population, we undertook a quantitative meta-analysis of all relevant observational and RCTs to determine safety and efficacy of Watchman 2.5 device vs. Amplatzer Amulet in patients undergoing LAAC procedure.

## Methods

We conducted this systematic review and meta-analysis in conformity with the established methods recommended by the Preferred Reporting Items for Systematic review and Meta-Analyses (PRISMA) and Cochrane guidelines [12, 13]. It was not required to obtain approval from the institutional review board as publicly available data was used in this study. This meta-analysis has been registered on PROSPERO (ID: CRD42023454974).

### Literature search strategy

We carried out a comprehensive and systematic search of the databases PubMed, Google Scholar, ScienceDirect, and ClinicalTrials.Gov for all studies that compared the safety and efficacy of Watchman and Amplatzer Amulet devices, from inception, till June 2023. There were no restrictions based on language, geographical location, and year of publication. A PRISMA search strategy was employed, utilizing Boolean Operators and PICO (Patient, Intervention, Control and Outcomes) criteria. We used the following MeSH terms including “Watchman”, “amplatzer”, “amulet”, “left atrial appendage occlusion” and “Left atrial appendage closure”. Moreover, to identify grey literature, reference list of relevant studies, Google scholar, and online libraries for example clinicaltrials.gov and pre-print servers like MedRvix.org (preliminary reports of work that have not been certified by peer review) were screened. These non-peer reviewed articles have not undergone this same level of scrutiny and are often published in magazines, newspapers, or other non-scholarly publications. While non-peer reviewed articles can still contain valuable information, they may not be as reliable or rigorously researched as peer-reviewed articles. A complete description of the search strategy used in each database is given in **S1 Table of S1 File**. Peer-reviewed articles published in English language were included in final analysis.

### Study selection and eligibility criteria

All duplicates were removed by exporting the retrieved articles to EndNote X9 Reference Manager (Clarivate Analytics, Philadelphia, Pennsylvania). Two independent researchers (A.M and E.A) then evaluated the remaining articles by reviewing the title and abstract. The full text was then assessed to ensure the inclusion of relevant articles. Any disagreements were resolved by consensus or discussion with another investigator (F.Y). In this meta-analysis studies that compared the Amplatzer Amulet and Watchman 2.5 devices directly, had a follow up time for 6 months or more, presented peri-procedural data, and had a sample size of 10 or more patients were chosen. The patients received anticoagulation therapy during study period and anti-thrombotic regimes varied across studies pooled including single or dual anti-platelet therapy, aspirin with or without oral anti-coagulation, or aspirin plus anti-platelet therapy. Studies that failed to reveal a comparable outcome measure for both devices were excluded from our review. Furthermore, single-arm studies, case reports, case series, and cohort studies with fewer than 10 patients, and studies that did not display appropriate safety and efficacy outcome data were also excluded.

### Data extraction

Participant and trial characteristics including name of author, year of study, study design, country of publication, sample size, age, and male population were extracted on an Excel sheet by two researchers (A.M and E.A). Additionally, data on safety and efficacy outcomes was also extracted. The safety outcomes of our study included device success, device-related thrombus, device embolization perioperative and at follow-up, perioperative pericardial perfusion events, and perioperative cardiac tamponade events. Efficacy outcomes were all-cause mortality perioperative and at follow-up, cardiovascular (CV) mortality at follow-up, stroke, major and minor bleeding events at follow-up, transient ischemic attack (TIA) in follow-up period, thromboembolic events in follow-up period, and peri-device leakage in perioperative period.

### Risk of bias and quality assessment

Two investigators (E.A and A.M) independently gauged the potential risk of bias of the RCTs using the Cochrane Risk of Bias (RoB 2.0) Tool [14]. Discussion with a third investigator (F.Y) was done in case of any disagreements. The Cochrane Risk of Bias tool and was used for the assessment of study quality. In the Cochrane tool, studies were assigned indicators on the basis of treatment allocation concealment and blinding, reporting on loss to follow-up, and providing outcome data on participants not included in the final analysis. For all other studies (observational studies), the Newcastle-Ottawa Scale was used to evaluate the quality of trials [15]. Studies were assigned” Good Quality” by a score of three–four in the selection realm, one–two in the comparability realm, and two–three in the result realm (for a total score of six–nine points).

### Statistical analysis

We performed the statistical analysis using Review Manager (V.5.4.1 Cochrane Collaboration, London, United Kingdom). The data were chiefly binary, using risk ratio (RR) to evaluate effects and calculate the 95% confidence interval (95% CI). Cochran Q statistic and I^2^ statistic [100% (Q −−− df) ∕Q] were used to measure heterogeneity. A random effect model was utilized to evaluate data. The funnel plot was not analysed to look for asymmetry to account for publication bias as no outcome had greater than ten studies. The heterogeneity across pooled studies was determined using Higgins I^2^ statistics. To assess for heterogeneity across the pooled studies Higgins I^2^ statistics was used, whereby a value of I^2^ = 25%-50% was considered mild, 50%-75% as moderate, and greater than 75% as severe heterogeneity [16]. A p-value of <0.05 was considered significant throughout.

## Results

### Study characteristics and baseline demographics

**Fig 1:**
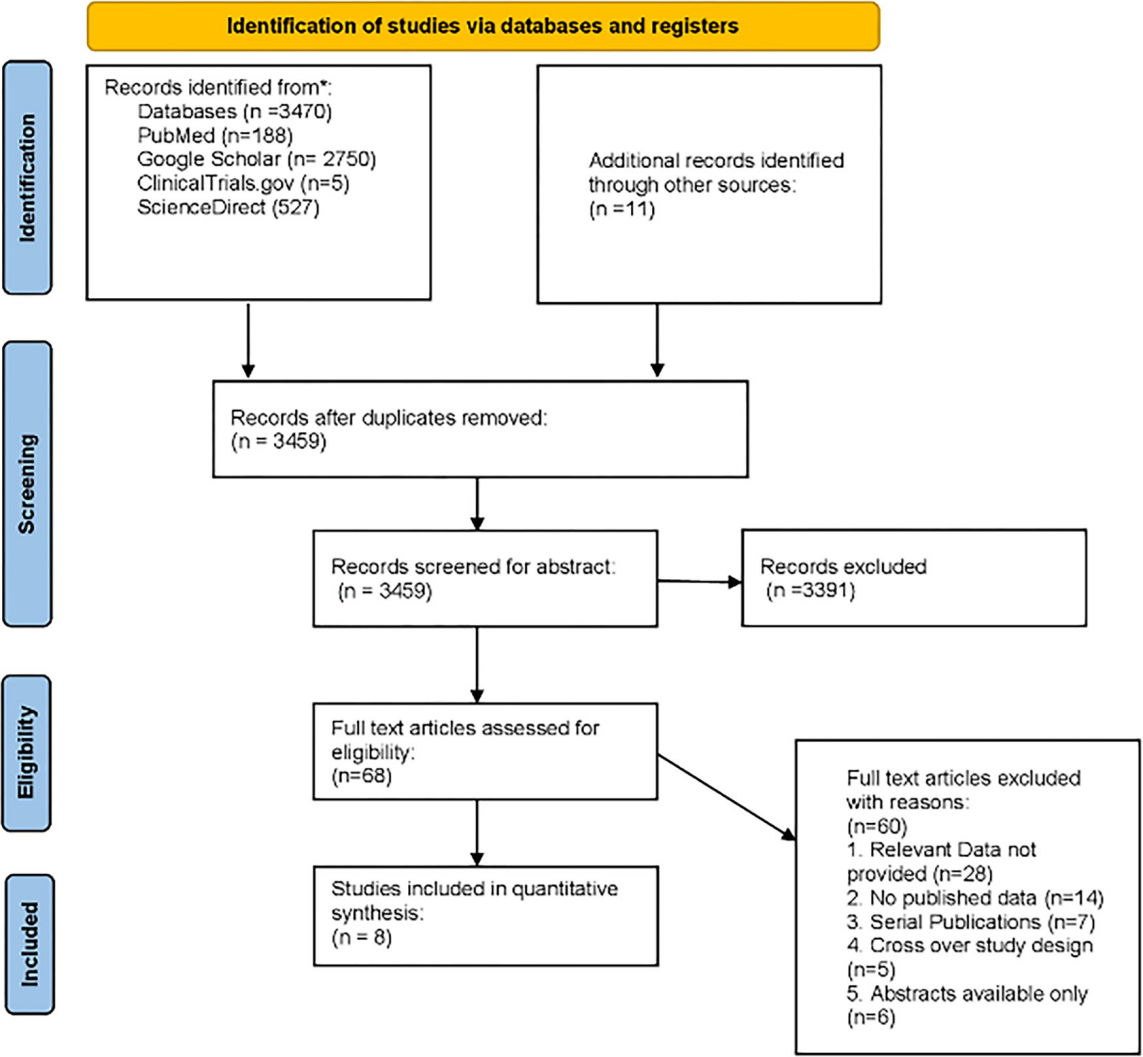
PRISMA flow diagram.

The PRISMA flow chart summarizes the search and study selection process in **Fig 1**. The search yielded a total of 3481 potential studies, and after screening eight studies, with a total of 3408 patients were included in our meta-analyses with a median follow-up of 12 months (IQR 6–12.3) [8, 11, 17–22], 1897 patients were in the Watchman group whereas 1511 patients were in the Amulet group. **Table 1** summarizes the general study characteristics of the included trials.

**Table 1:** General characteristics of the included studies.

*Study*	*Country*	*Study design*	*Sample size*		*Male*, *n (%)*		*Age*		*CHA*_*2*_ *DS*_*2*_ *-VASc score*		*HAS-BLED score*		*Previous stroke/TIA*, *n (%*		
			*Watchman 2*.*5*	*Amulet*	*Watchman 2*.*5*	*Amulet*	*Watchman 2*.*5*	*Amulet*	*Watchman 2*.*5*	*Amulet*	*Watchman 2*.*5*	*Amulet*	*Watchman 2*.*5*	*Amulet*	*Follow-up (months)*
Fastner, 2018	Germany	RCS	154	35	105 (68.2)	22 (62.9)	75.2 ± 2.8	77.1 ± 9.7	4.5 ± 0.1	4.0 ± 1.4	3.6 ± 0.2	3.7 ± 1.0	-	-	6
Kefer, 2018	Belgium	PCS	139	144	94 (67.6)	82 (56.9)	75 ± 8	77 ± 8	4.5 ± 1.7	4.7 ± 1.5	3.1 ± 1.1	3.1 ± 0.9	57 (41)	71(49	12.3
Chen, 2019	Germany	PCS	36	74	26 (72.2)	49 (66.2)	75.3 ± 8.6	76.0 ± 7.9	3.6 ± 1.5	3.9 ± 1.5	3.8 ± 1.0	3.9 ± 1.9	6 (16.7)	16 (21.6)	6
Lakkireddy 2021	USA	RCT	944	934	579 (61.3)	549 (58.8)	75.1 ± 7.6	75.0 ± 7.6	4.7 ± 1.4	4.5 ± 1.3	3.3 ± 1.0	3.2 ± 1.0	188 (19.9	168 (18.0	18
Mansour, 2022	France	RCT	25	26	19 (76.0)	20 (76.9)	76 ± 6.9	75 ± 7.4	3.9 ± 1.27	3.9 ± 1.16	4.2 ± 0.9	4.1 ± 1.2	12 (48)	12 (46)	12
Radinovic, 2021	Italy	RCS	97	92	49 (50.5)	55 (59.8)	73.6 ± 8.3	71.6 ± 9.9	3.2 ± 1.3	3.5 ± 1.2	3.8 ± 1.6	4.1 ± 1.5	10 (10.9)	7 (7.2)	12
Saad, 2021	Germany	RCS	113	113	70 (61.9)	70 (61.9)	76 ± 8	78 ± 6	4 (3−5)	4 (3−5)	3 (2−3)	3 (2−3)	10 (9	7 (6)	12
Kretzler, 2022	Germany	PCS	389	93	256 (66)	56 (58)	75.1 ± 8.5	74.4 ± 8.8	4.04 ± 1.546	4.22 ± 1.548	3.62 ± 1.105	3.34 ± 1.238	117 (30.1)	25 (26.9)	1.5

**Abbreviations:** RCS: Retrospective Cohort study; PCS: Prospective Cohort Study; RCT: Randomized Controlled Trial

### Quality assessment and risk of bias

Two independent reviewers (E.A and A.M) assessed the risk of bias by using Newcastle Ottawa scale for observational studies and RoB-2.0 for RCTs. For all studies, one reviewer (E.A) extracted all the data and assessed the risk of bias, while a second reviewer (A.M) cross-checked the information for completeness and accuracy. Based on the quality assessment scale, three cohort studies were rated as ‘Good’, and three cohort studies were rated as ‘Fair’ quality. Using a quality assessment tool for RCTs, an overall low risk of bias was found for 2 included RCTs. A low risk of bias was reported in all domains as the procedure, outcomes and analysis were adequate in the studies. Detailed tables of Quality and Risk of Bias Assessment results are presented in the supplementary material (**S1, S2 Figs and S2 Table in S1 File**).

### Safety outcomes (device success, device-related thrombus, pericardial effusion, device embolization, and cardiac tamponade)

**Fig 2:**
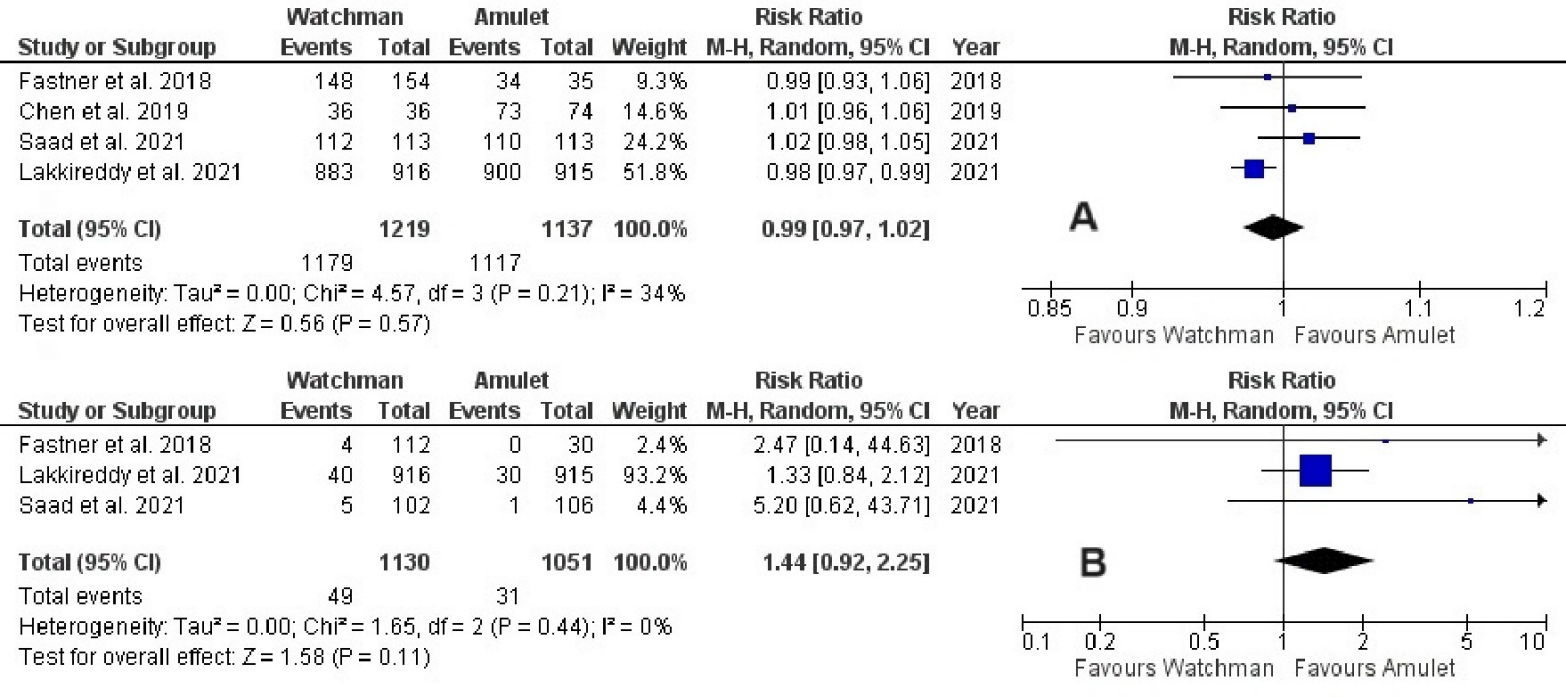
(A) Device success and Figure (B) Device-related thrombus between Watchman and Amulet devices.

**Fig 3:**
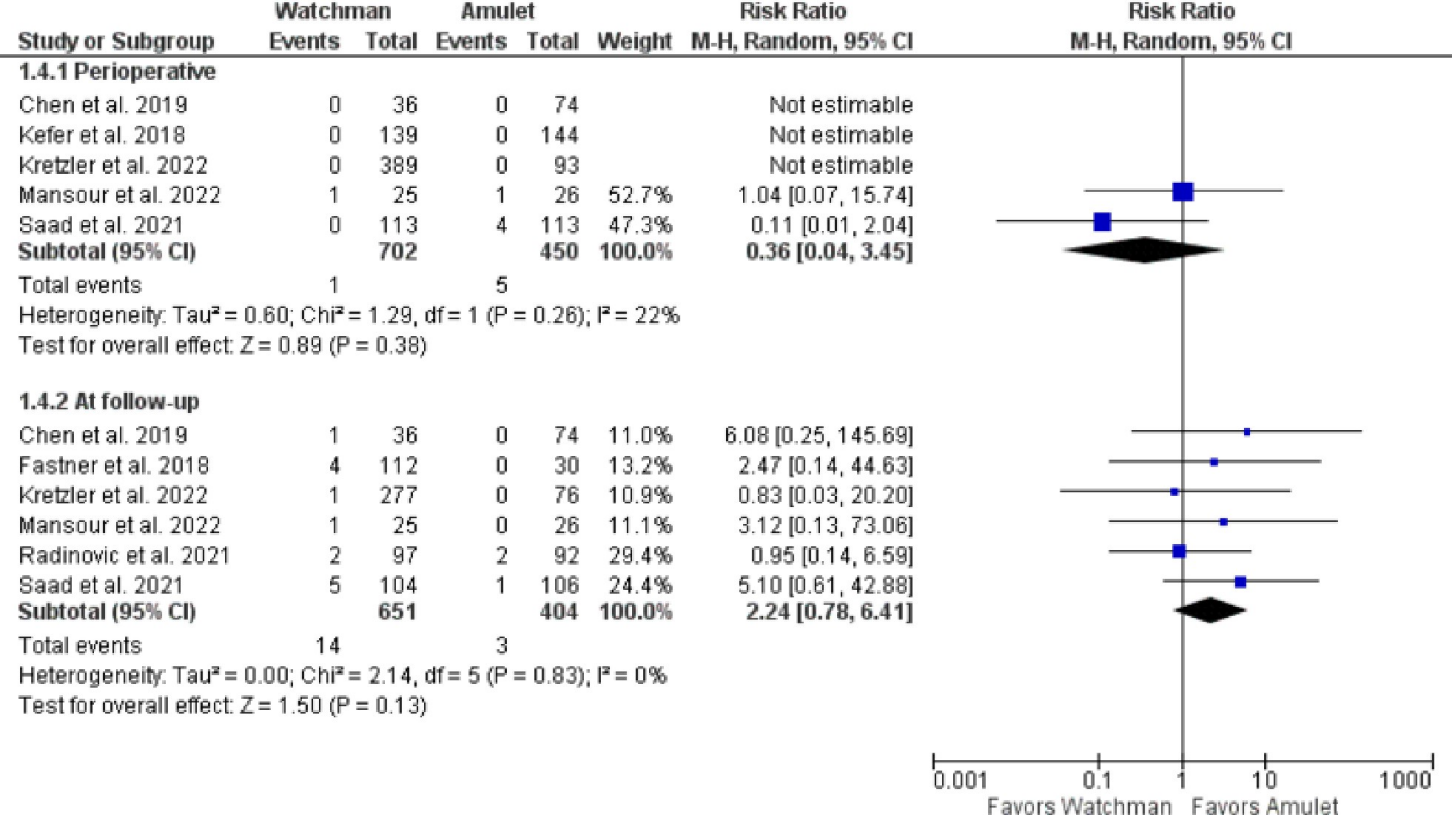
Perioperative and at follow-up incidence of device embolization.

**Fig 4:**
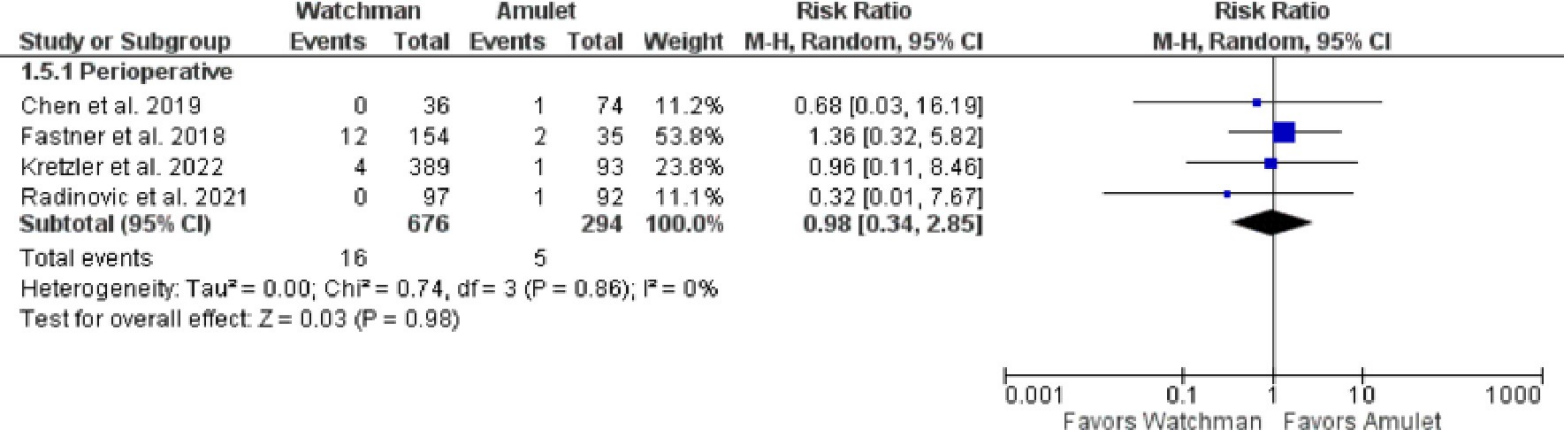
Perioperative pericardial effusion events.

**Fig 5:**
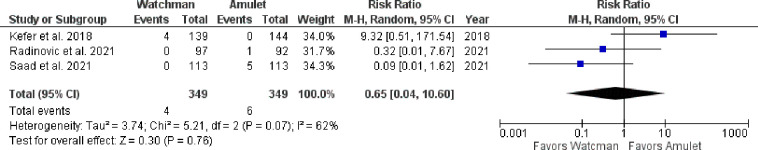
Perioperative cardiac tamponade events.

Device success was non-significantly reduced in Watchman 2.5 group when compared with Amulet (RR 0.99, 95% CI 0.97–1.02, p = 0.57; I^2^ = 34%) (Fig 2A). In contrast, device-related thrombus was non-significantly increased in Watchman 2.5 group in comparison to Amulet (RR 1.44, 95% CI 0.92–2.25, p = 0.11; I^2^ = 0%) (Fig 2B). There was also no significant difference between the devices in terms of device embolization in the perioperative (RR 0.36, 95% CI 0.04–3.45, p = 0.38; I^2^ = 22%) and follow-up (RR 2.24, 95% CI 0.78–6.41, p = 0.13; I^2^ = 0%) periods (**Fig 3**). Likewise, in relation to pericardial effusion there was no significant difference between the devices perioperatively (RR 0.98, 95% CI 0.34–2.85, p = 0.98; I^2^ = 0%) (**Fig 4)**. Similarly, cardiac tamponade was statistically similar in both the groups in perioperative period (RR 0.65, 95% CI 0.04–10.60, p = 0.76; I^2^ = 62%) (**Fig 5**).

### Efficacy outcomes (all-cause mortality, CV mortality, stroke, thromboembolic, TIA, peri-device leakage, major and minor bleeding)

**Fig 6:**
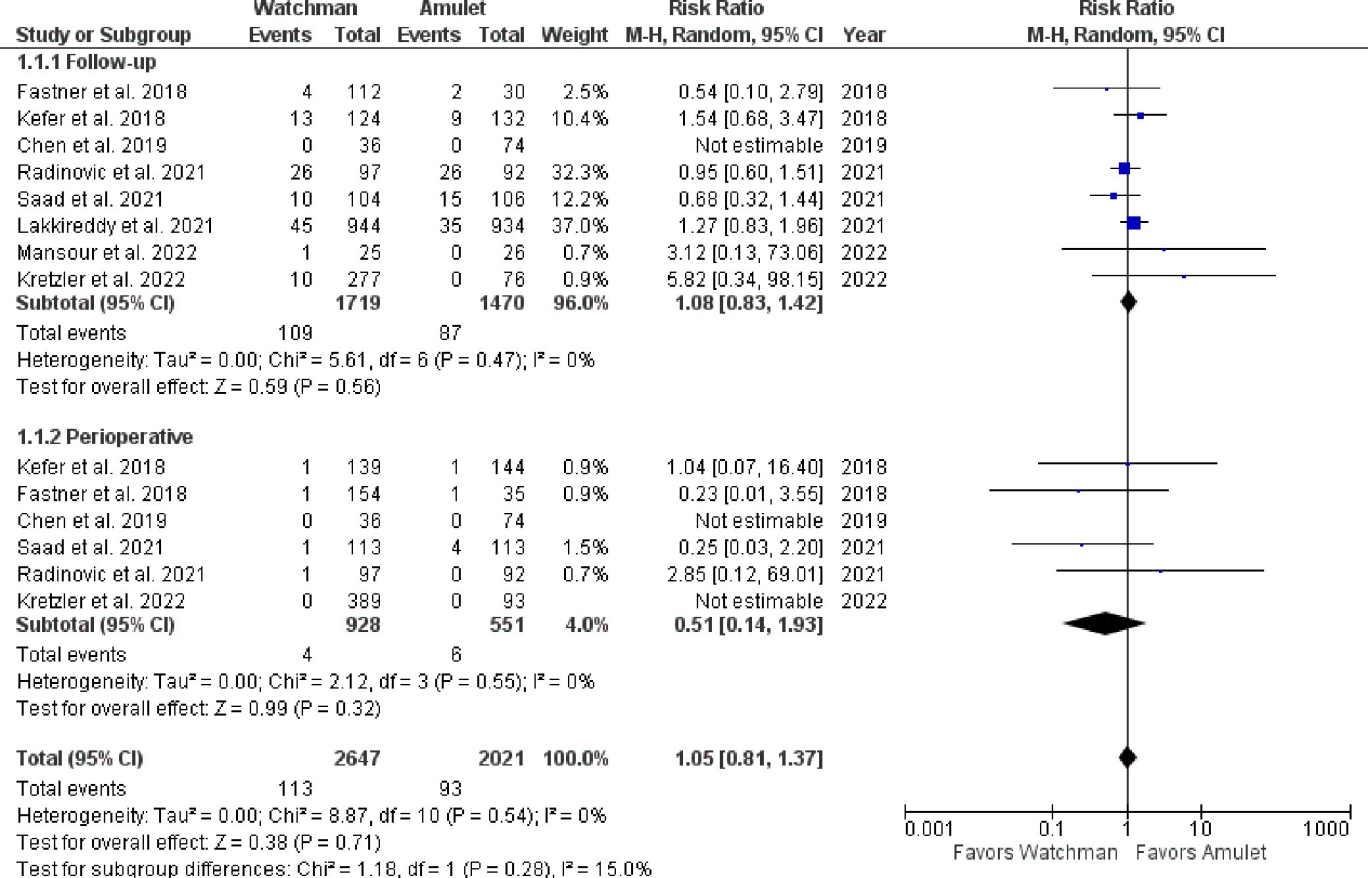
Perioperative and at follow-up all-cause mortality.

**Fig 7:**
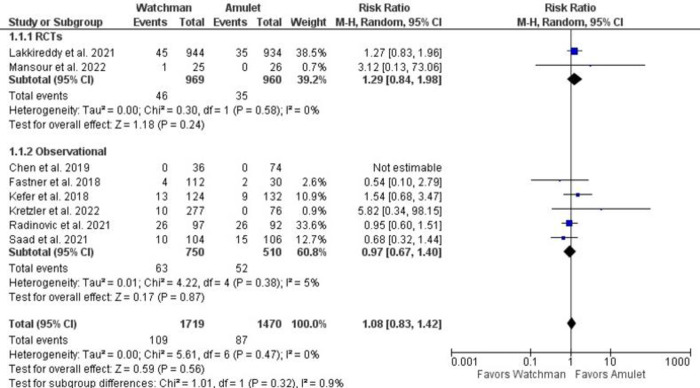
Subgroup analysis of all-cause mortality based on study type.

**Fig 8:**
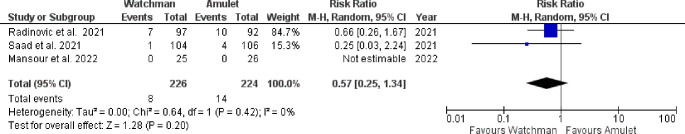
CV mortality events between Watchman and Amulet devices.

**Fig 9:**
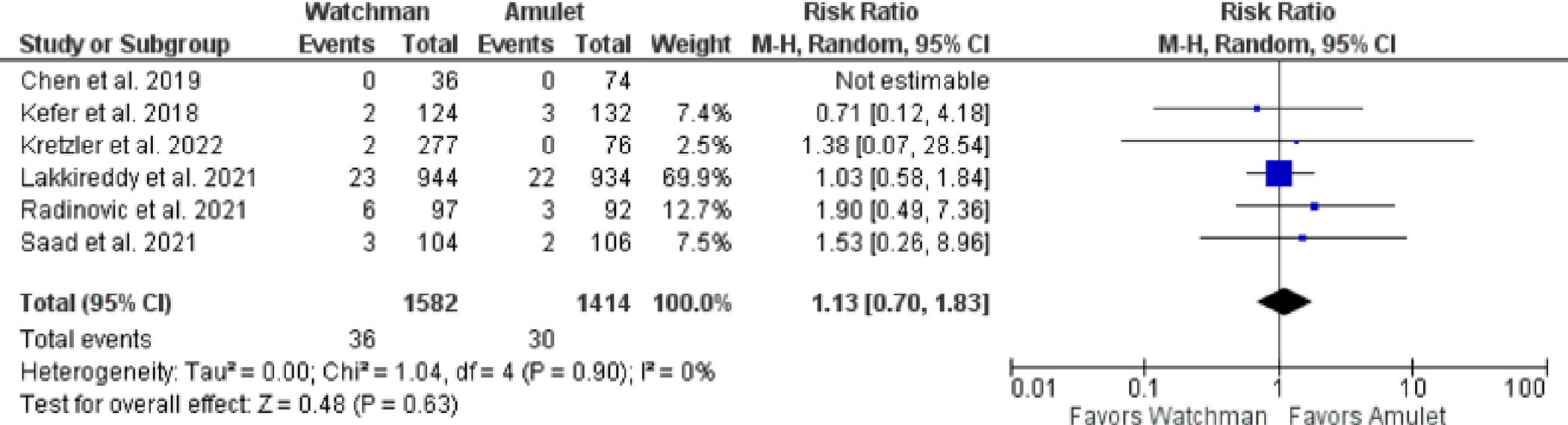
Incidence of stroke at follow-up.

**Fig 10:**
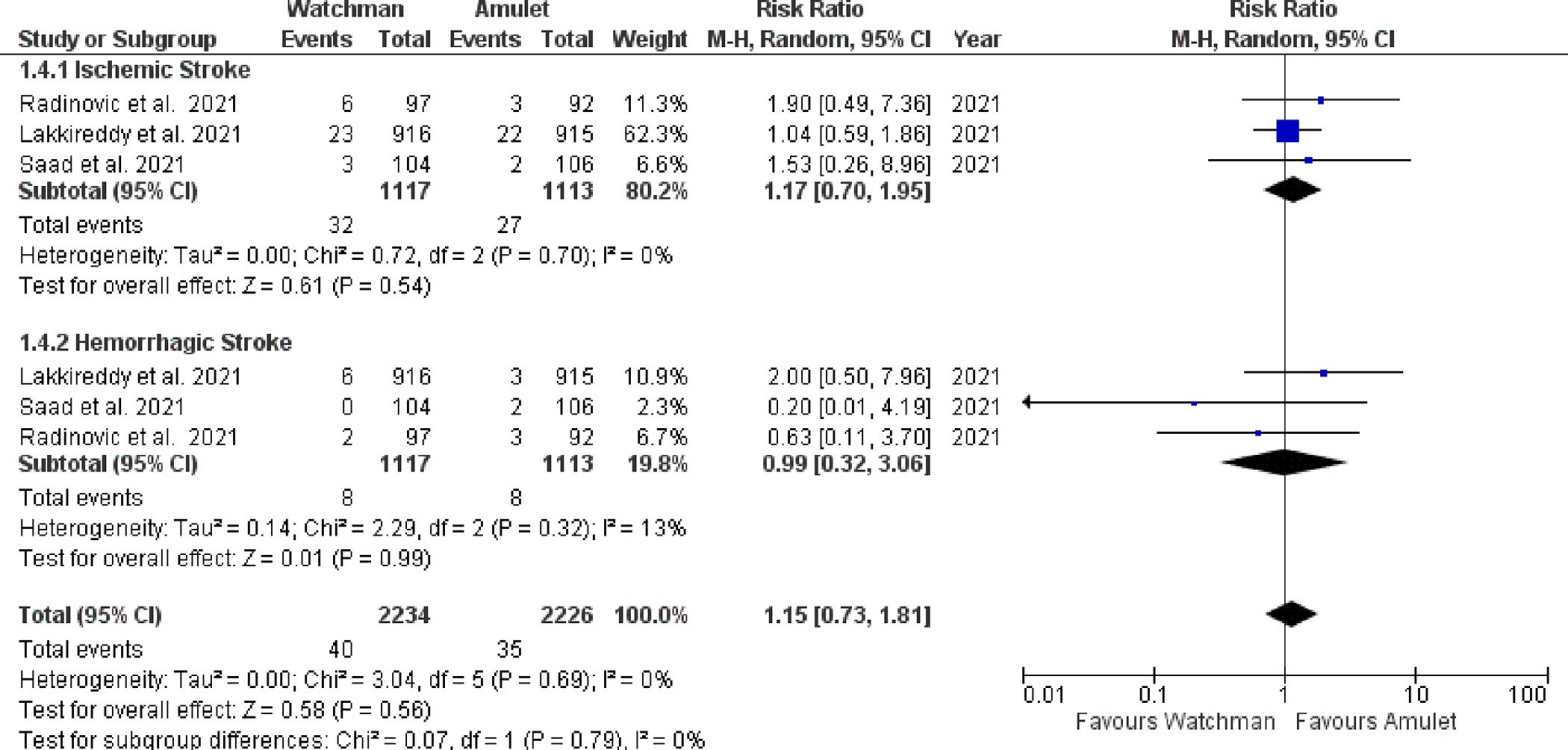
Sub-group analysis based on type of stroke between two devices.

**Fig 11:**
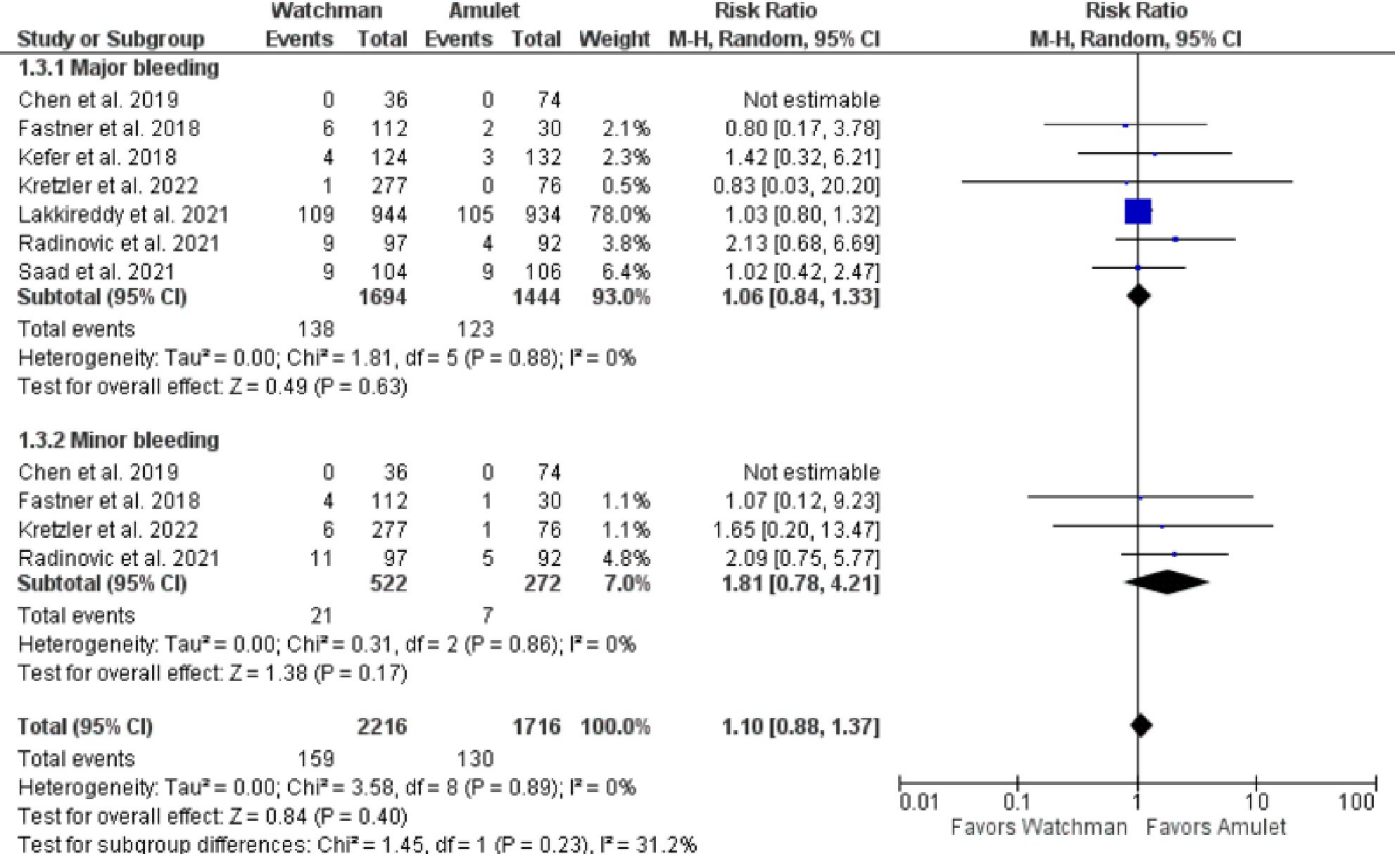
Major and minor bleeding at follow-up.

**Fig 12:**
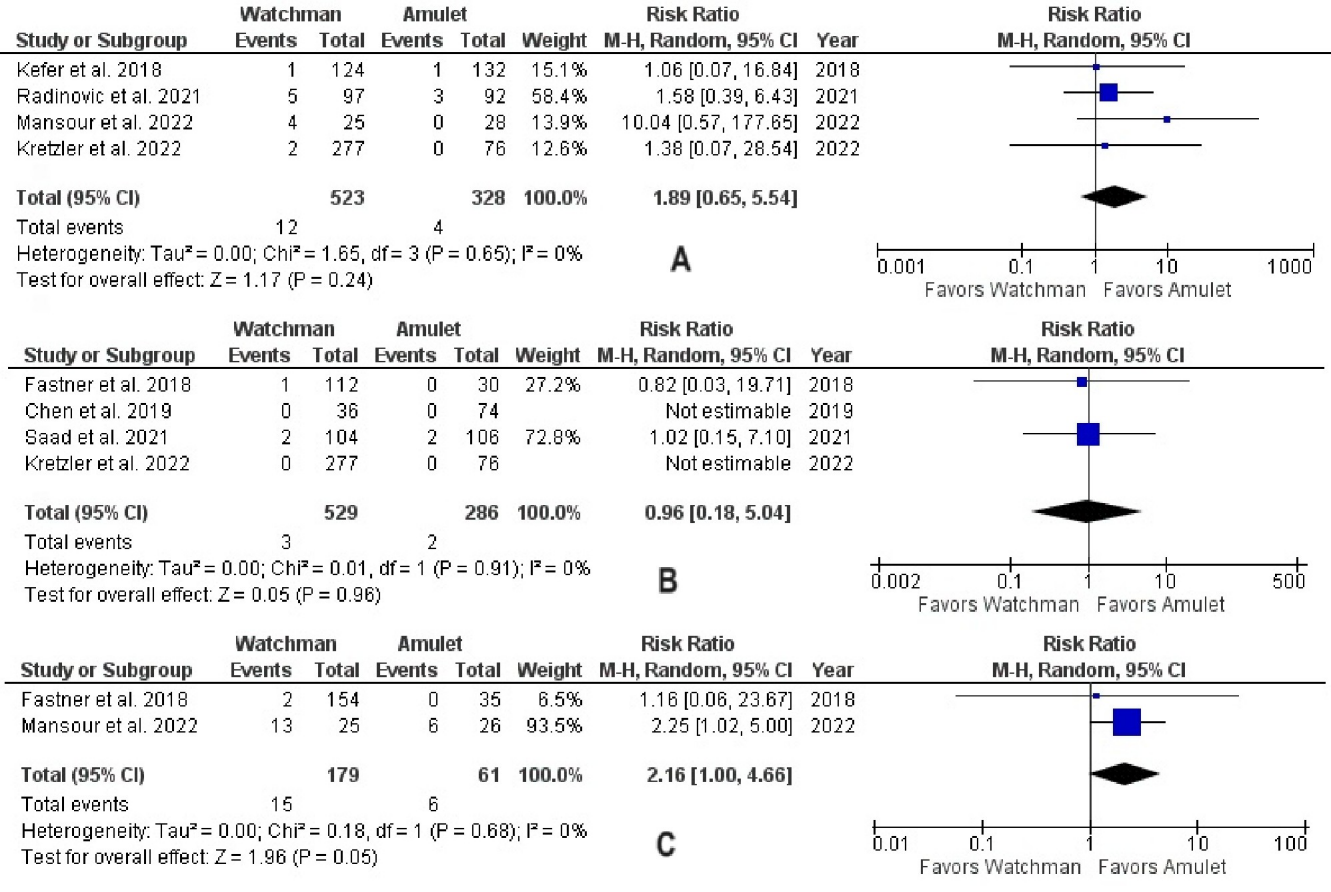
(A) Transient ischemic attack in follow-up period, (B) thromboembolic events in follow-up period, and (C) peri-device leakage in perioperative period.

There was no significant difference observed between Watchman and Amulet devices for perioperative (RR 0.51, 95% CI 0.14–1.93, p = 0.32; I^2^ = 0%) and follow-up (RR 1.08, 95% CI 0.83–1.42, p = 0.56; I^2^ = 0%) all-cause mortality, as shown in **Fig 6**. Furthermore, upon subgroup analysis we found out that there was no significant difference in all-cause mortality between RCTs and observational studies (p = 0.32; I^2^ = 0.9%) (**Fig 7**). CV mortality was non-significantly reduced in Watchman group when compared with Amulet (RR 0.57, 95% CI 0.25–1.34, p = 0.20; I^2^ = 0%) **(Fig 8).** The Amulet device was not superior to the Watchman device in terms of stroke at follow-up (RR 1.13, 95% CI 0.70–1.83, p = 0.63; I^2^ = 0%), as there was no significant difference between them (**Fig 9)**. Upon sub-group analysis for stroke outcome, there was no significant risk of ischemic (RR 1.17, 95% CI 0.70–1.95, p = 0.54; I^2^ = 0%) and haemorrhagic stroke events (RR 0.99, 95% CI 0.32–3.06, p = 0.99; I^2^ = 13%) between two devices **(Fig 10).** Furthermore, at follow-up, there was no significant difference in major (RR 1.06, 95% CI 0.84–1.33, p = 0.63; I^2^ = 0%) and minor bleeding events (RR 1.81, 95% CI 0.78–4.21, p = 0.17; I^2^ = 0%) between the two devices (**Fig 11**). At follow-up, no difference was observed between transient ischemic attack events (RR 1.89, 95% CI 0.65–5.54, p = 0.24; I^2^ = 0%), as shown in **Fig 12A**. No difference between the Watchman and Amulet groups was seen in thromboembolic events in follow-up period (RR 0.96, 95% CI 0.18–5.04, p = 0.96; I^2^ = 0%) (**Fig 12B)** and peri-device leakage in perioperative period (RR 2.16, 95% CI 1.00–4.66, p = 0.05; I^2^ = 0%) (**Fig 12C)**.

## Discussion

This systematic review and meta-analysis aimed to investigate the safety and efficacy of the Amplatzer Amulet and Watchman 2.5 devices for LAAC. No significant difference was observed in the outcome of device embolization (DE) in either the perioperative or follow-up periods between the two devices. Likewise, pericardial effusion and cardiac tamponade were statistically similar perioperatively in both devices. Perioperative and follow-up all-cause mortality were also comparable in both Amulet and Watchman 2.5. Furthermore, there was no significant difference in perioperative peri-device leakage and follow-up stroke, major and minor bleeding events, transient ischemic attack, and thromboembolic events in either of the two groups. The results of our analysis concord with a previous meta-analysis conducted by Zhu et al. comprising of seven studies involving 2926 patients [23]. The study reported no significant differences in the safety and efficacy of both devices. Our meta-analysis differs from Zhu et al. such that we included multi-center data of 482 patients from the ORIGINAL registry reported in a study conducted by Kretzler et al. [11]. The addition of this study to our analysis reduced the overall complication rates in both groups to a significant extent, showing that this multi-center study was carried out at more experienced centers with lower failure and complication rates.

In terms of safety, our results demonstrated no significant difference in the occurrence of DE during either the perioperative or follow-up periods. However, there is a numerical trend suggesting a slightly higher occurrence of DE in the perioperative period in the Amulet group compared to Watchman group. Conversely, DE during the follow up time, although still statistically insignificant, was numerically higher in Watchman group compared to Amulet group. The majority of peri-procedural DE events in the Amulet group in our analysis were reported by Saad et al. [22]. The authors proposed that the higher number of peri-procedural complications observed in the Amulet group in their study could be attributed to early implantation experience with the Watchman device compared to the Amulet device, which was introduced at their center much later. A retrospective analysis conducted by Lakkireddy et al. found a 2% incidence rate of DE when reviewing the Watchman, Amplatzer Cardiac Plug and Amulet devices for around 5000 LAAC procedures [24]. Of these, 30% occurred with the Watchman device and 11% with the Amulet. The author reported DE to have occurred more commonly during the postoperative period than the intra-operative period (61% vs 39%, p = 0.06). The author further stated that device/left atrial appendage (LAA) size mismatch was the most commonly identifiable factor associated with DE. Hence, it can be inferred, that perhaps the occurrence of DE is correlated with the size of the device compared to the anatomy of the left atrium (LA), rather than the type of device used itself. Consequently, appropriate measurements of the LA morphology and corresponding device sizing may prevent DE in both Watchman 2.5 and Amulet devices.

Similarly, the rate of pericardial effusion and cardiac tamponade were statistically similar perioperatively in both devices. In a prospective study of 1088 patients using the Amulet occluder, the rate of pericardial effusion requiring surgical intervention was as low as 1.3% (9). Correspondingly, according to several studies, the rates of these complications with the Amulet device are minimal [25]. However, for the Watchman device, data regarding rates of pericardial effusion remains inconsistent. In the Protect AF trial, 4.5% of participants undergoing the procedure with the Watchman device suffered from pericardial effusion whereas in the CAP registry and the PREVAIL trial, the rates were as low as 2.2% [5, 6, 26]. It has been suggested that these complications have lessened over time with the help of experienced operators with adequate training in performing the implantation procedure [27]. Nevertheless, according to our analysis, no significant difference was observed for either pericardial effusion or cardiac tamponade in either of the two devices.

Our pooled analysis demonstrated no significant differences in the rates of major and minor bleeding events between both groups. However, despite reasonable safety and efficacy profiles of both devices, the incidence of major bleeding at follow-up was relatively high in both groups. Aminian et al. analyzed the two-year results of the Amplatzer Amulet Observational Post-Market Study and found that prior stroke and pre-LAAC major bleeding were independent predictors of major bleeding during follow-up [28]. Similarly, a specific analysis of 318 patients with a prior major bleeding event receiving the Watchman LAAC device in the EWOLUTION trial demonstrated higher rates of non-procedural bleeding events at follow-up [29]. Furthermore, in a propensity-adjusted analysis of the EWOLUTION trial, early discontinuation of dual antiplatelet therapy (DAPT) post-procedure was associated with lower bleeding rates compared to those with late discontinuation (1.1% vs 3.5%). Future investigations will benefit from assessing the impact of high bleeding risk profiles and the use of APT/oral anticoagulants post-procedure on major bleeding events after an LAAC procedure using the Watchman and Amulet devices.

According to several trials and registries, both the Watchman 2.5 and Amulet devices have demonstrated clinical effectiveness in the prevention of stroke, mortality and ischemic events [25, 30–32]. Our overall pooled analysis showed no significant differences in the rates of stroke, mortality, and transient ischemic attack between both devices for LAAC. A subgroup analysis of RCTs and observational studies for the outcome of mortality also established no significant differences between both groups.

The results of this meta-analysis reported thromboembolic events at follow-up and peri-device leakage in the perioperative period to be comparable for both devices. A systemic review conducted by Lempereur et al. investigated device-associated thrombus (DAT) formation after LAAC using the Watchman, Amplatzer Cardiac Plug and Amulet devices in a total of 30 studies [33]. According to their analysis, thrombus formation most frequently occurred at the proximal connector pin, the only area of the device uncovered by nitinol or a permeable polyester fabric. DRT is also associated with a three-fold higher risk for systemic embolism and stroke [34]. Taking these risk factors into mind, the Amulet device and newer generation Watchman devices have lower profile proximal screws to reduce the risk of thrombus formation [35]. Hence, both devices can be considered safe to use with minimal risk of thromboembolic events associated with them.

The incidence of peri-device leakage, albeit statistically insignificant, was numerically higher in the Watchman group compared to Amulet. These results should be interpreted with caution as only two out of the eight studies included in our analysis reported on this outcome. The Amulet device consists of a dual seal mechanism with an effective occlusion strategy that overcomes the limitations of a single seal mechanism (short LAA length, proximal lobes near the ostium, and very large ostia) [36]. Hence, this could explain why, in our analysis, the Amulet device was associated with a lower numerical rate of peri-device leakage compared with the Watchman device. Furthermore, the malalignment of the device to the left appendage ostium has also been suggested as a cause of leakage in some studies [37, 38]. It is imperative for investigators to conduct robust clinical trials to further assess the risk of this outcome in both devices.

It is beneficial to mention that most studies included in this analysis utilized similar implantation techniques to conduct both procedures. Assessment of the LAA and its anatomy was mostly conducted using transoesophageal echocardiography (TEE) in most cases, followed by fluoroscopy and angiography, respectively. Most procedures were conducted under general anaesthesia and a transseptal puncture was performed in almost every case under the guidance of TEE or fluoroscopy. The size of the device implanted varied across studies according to the anatomy of the LA. As mentioned previously, a mismatch between the LAA size compared to the device was suggested to be correlated with the occurrence of DE in several studies.

It is also crucial to mention that while most studies employed comparable procedural techniques, a significant variation was observed in the antithrombotic regimen administered post-procedure. In some studies, antithrombotic therapy was tailored according to several features such as bleeding risk, success of device seal and extent of leakage, device embolization and atrial fibrillation ablation before the procedure. For instance, in the study conducted by Mansour et al., all patients had contraindications to long term antithrombotic therapy owing to gastrointestinal and intracranial bleeding, serious epistaxis, and recurrent falls. Hence, future investigations should assess the effect of the type and extent of antithrombotic therapy on long-term complications in patients undergoing LAAC procedures using the Watchman and Amulet devices. Details of the implantation techniques deployed, and the antithrombotic regimens administered in each investigation are mentioned in S3 Table in S1 File.

Overall, the findings of this meta-analysis suggest that the Watchman 2.5 and Amplatzer Amulet devices are comparable in terms of safety and efficacy for left atrial appendage occlusion in patients with NVAF. These devices offer a viable alternative to long-term oral anticoagulation for stroke prevention in patients who are contraindicated for anticoagulant therapy or have poor compliance with oral anticoagulants. Despite the valuable insights gained from this study, there are certain limitations that should be acknowledged. First, the available literature on the comparison of Watchman 2.5 and Amplatzer Amulet devices is limited, and the number of studies included in the meta-analysis was relatively small. This may have affected the statistical power to detect potential differences between the devices. Second, there may have been variations in patient selection criteria and procedural techniques across the included studies, which could have influenced the outcomes. Additionally, the follow-up duration across the studies was not consistent, which may have influenced the long-term outcomes. To uncover disparities in the success of Amplatzer Amulet and Watchman, we also included data from several countries, which could have contributed to the heterogeneity of analysis. Subgroup analysis based on geographical location, low, and high-risk stroke groups could not be conducted owing to inadequate sample size. Furthermore, this meta-analysis was a study-level analysis, and did not analyse patient-level data.

## Conclusion

In conclusion, this systematic review and meta-analysis provide evidence that there is no significant difference in safety and efficacy outcomes between the Watchman 2.5 and Amplatzer Amulet devices for left atrial appendage occlusion in patients with non-valvular atrial fibrillation. Both devices appear to be effective and safe alternatives to oral anticoagulation for stroke prevention in this patient population. However, it is essential to consider individual patient characteristics and preferences when selecting the most appropriate device for left atrial appendage occlusion. Further research with larger sample sizes and longer follow-up periods is warranted to validate these findings and provide more robust evidence for clinical decision-making.

## Supporting information

S1 File Electronic supplementary material–Watchman vs. Amulet. (DOCX)
