# Supplementary material for: Safety and efficacy of percutaneous Watchman 2.5 device versus Amplatzer Amulet for left atrial appendage closure in patients with non-valvular atrial fibrillation: A systematic review and study-level meta-analysis
Source: PLoS One. 2024 Feb 14;19(2):e0295804. doi: 10.1371/journal.pone.0295804 (PMC10866506; doi:10.1371/journal.pone.0295804)

**Electronic Supplementary Material**

**Supplementary Table 1: Search strategy used in each database searched.**

| Database | Search Strategy | Articles retrieved |
| --- | --- | --- |
| Pubmed | "watchman"[All Fields] AND ("amulet"[All Fields] OR "amulets"[All Fields] OR ("amplatz"[All Fields] OR "amplatzer"[All Fields])) AND (("left"[All Fields] AND ("atrial appendage"[MeSH Terms] OR ("atrial"[All Fields] AND "appendage"[All Fields]) OR "atrial appendage"[All Fields]) AND ("closure"[All Fields] OR "closure s"[All Fields] OR "closures"[All Fields])) OR ("left"[All Fields] AND ("atrial appendage"[MeSH Terms] OR ("atrial"[All Fields] AND "appendage"[All Fields]) OR "atrial appendage"[All Fields]) AND ("dental occlusion"[MeSH Terms] OR ("dental"[All Fields] AND "occlusion"[All Fields]) OR "dental occlusion"[All Fields] OR "occlusion"[All Fields] OR "occlused"[All Fields] OR "occlusions"[All Fields] OR "occlusive"[All Fields] OR "occlusives"[All Fields])) OR "LAAC"[All Fields]) | 188 |
| Google Scholar | (Watchman) AND (amplatzer) AND (left atrial appendage occlusion OR LAAC) | 2750 |
| ScienceDirect  ClinicalTrials.gov | (watchman) AND (amulet) AND (left atrial appendage OR LAAC)  (Watchman) AND (amulet) AND (left atrial appendage) | 527  5 |

| STUDY ID | SELECTION ^a^ | | | | COMPARABILITY ^b^ | OUTCOME ^c^ | | | QUALITY ASSESSMENT BASED ON AHRQ ^d^ |
| --- | --- | --- | --- | --- | --- | --- | --- | --- | --- |
|  | Representativeness of the exposed cohort | Selection of non-exposed cohort | Ascertainment of exposure | Demonstration that outcome of interest was not present at start of study | Comparability of cohorts on basis of design or analysis controlled for confounders | Assessment of outcome | Adequacy of duration of follow up | Adequacy of completeness of follow up |  |
| Fastner et al. 2018 | * | * | * |  | * | * | * |  | Fair |
| Kefer et al. 2018 | * | * | * | * | ** | * | * | * | Good |
| Chen et al. 2019 | * | * | * | * | * | * | * | * | Good |
| Saad et al. 2021 | * | * | * | * | ** | * | * | * | Good |
| Radinovic et al. 2021 | * | * | * |  | * |  | * | * | Fair |
| Kretzler et al. 2022 | * | * | * | * | * | * |  | * | Fair |

**Supplementary Table 2: Quality Assessment for included cohort studies.**

^a,b,c:^ The higher the number of asterisk (*), the better quality of a given criterion. The maximum asterisks possible for each domain are selection = 3, comparability =2, outcome = 3. ^d:^ The Newcastle Ottawa Scale for Cohort studies converted to AHRQ standards (good, fair, poor). AHRQ: Agency for Healthcare Research and Quality.

**Supplementary Figure 1: ROB-2 Assessment for RCTs**


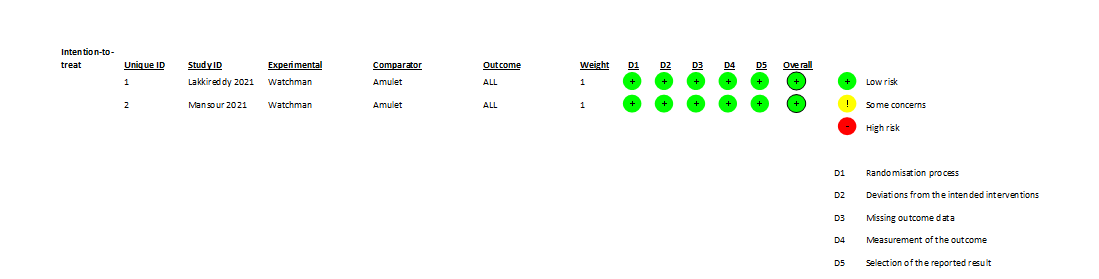


**Supplementary Figure 2: As percentage (intention-to-treat) for included RCTs**

**Supplementary Table 3: Device implantation procedure and antithrombotic regimen post-procedure in the pooled studies**

| **Study** | **Implantation procedure** | **Antithrombotic regimen post-procedure** |
| --- | --- | --- |
| Fastner, 2018 | Procedure performed under conscious sedation and guided by TEE, angiography and fluoroscopy. Device selection based on LA anatomy. After release of device, venous access site sealed via Z-suture or ProGlide. Device leaks observed via TEE. | Centre 1; antithrombotic regimen individualized  Centre 2; aspirin and clopidogrel for 6 months followed by lifelong aspirin.  Combined (first 6 months); 94.6% and 100% on DAPT, 1.4% and 0% on OAC plus clopidogrel, in the Watchman and Amulet group, respectively. |
| Kefer, 2018 | General anesthesia administered. TEE and fluoroscopic guidance used. Sheath advanced from femoral vein to LA after a transseptal puncture. Heparin is also administered along with antibiotics. LAA angiogram performed on several projections. After device is implanted, stability tests performed using TEE. Residual flow assessed via contrast angiogram. | Low dose aspirin and clopidogrel for 1 month in both groups. A total of 81 patients discharged on DAPT, 3 on SAPT, and 5 on OAC. |
| Chen, 2019 | Boluses of midazolam for deep sedation. Continuous infusion of 1% propofol. A single transseptal puncture used after which one 8-Fr sheath inserted into LA. Heparin (80-100 IE/Kg of body weight) provided. Transseptal sheath exchanged with LAAO delivery sheath under guidance of TEE and fluoroscopy. Activated clotting time 250-300 s. Device implantation performed using right anterior oblique 30 degrees plus caudal or cranial view. Angiography or TEE used to assess degree of sealing. | Amulet group; 3-6 months of DAPT followed by SAPT. 90.5% of patients discharged on DAPT.  Watchman group; 6 weeks of OAC and aspirin. If seal is appropriate and no device thrombosis detected, DAPT and clopidogrel/aspirin given till 6 months. Aspirin continued for life. |
| Lakkireddy, 2021 | Patients received aspirin day before implant (81-100 mg). Either local or general anesthesia used. Guidance via TEE or fluoroscopy. Contrast injection used to compare size of LAA with that of device. LAA depth from LAA orifice must be 10-12 mm. procedure terminated if requirements not met. TEE used to assess degree of sealing. | Amulet group; patients discharged on either aspirin/clopidogrel (jet <5mm) or aspirin/OAC (jet>5mm). OAC stopped at 45-day follow-up if residual jet leak <5mm.  Watchman group; patients discharged on aspirin and warfarin.  *Clopidogrel continued for 6 months and aspirin for lifetime |
| Mansour, 2021 | General anesthesia used. TEE used to obtain LAA and ostium size and depth at 0, 45, 90 and 130 degrees. In mid-esophageal views. Right femoral vein used as access site for passing catheters. Under TEE and fluoroscopy guidance infero-posterior transseptal puncture was obtained. Intravenous normal saline solution run if LAP<11mmHg. LAA reassessed via TEE and CCTA. Device stability ensured using tug test and measurement of device leakage. | All patients contraindicated to long term antithrombotic therapy. Depending on bleeding risk, patients were discharged on aspirin with or without OAC. patients with decreased bleeding risk and significant device leakage discharged on OAC for 2 months. |
| Radinovic, 2021 | Baseline TEE performed. LAAO performed according to individual device instructions. TEE used to assess post-procedural complications (device leakage and thrombi) | Device’s instructions for use followed by 47% of patients. Tailored therapy in case of increased bleeding risk; LMWH 13%, SAPT 6%, triple therapy 5%, no therapy 3%. |
| Saad, 2021 | LAA anatomy assessed using TTE or TEE. Procedure performed under sedation by midazolam, fentanyl and propofol. Heparin administered and clotting time kept above 300 seconds. TEE or fluoroscopy guidance used for transseptal puncture with an SL1 sheath. A pigtail catheter inserted into LAA over a soft J-tipped 0.035-inch wire. Measurement of LAA done in right anterior oblique caudal and cranial projections. Closure assessed via TEE and angiography. Tug test used to determine optimal positioning of device | 60.7% and 79.5% discharged on DAPT in the Watchman and Amulet group, respectively. |
| Kretzler, 2022 | NR | At 6-week follow-up, 1.9% of patients in the Amulet group and 2.6% in the Watchmen group on DAPT (aspirin and a P2Y2 inhibitor). 7.9% and 2.6% in the Amulet and Watchman group, respectively, on LMWH and antiplatelet therapy |

**Abbreviations:** TEE: transesophageal echocardiography; DAPT: Dual antiplatelet therapy; OAC: oral anticoagulants; SAPT: single antiplatelet therapy; LAP: Left atrial pressure; CCTA: Coronary computed tomography angiography; LMWH: low molecular weight heparin; TTE: transthoracic echocardiography

**Supplementary Table 4: Preferred Reporting Items for Systematic Reviews and Meta-analyses (PRISMA) checklist**

| **Section and Topic** | **Item #** | **Checklist item** | **Location where item is reported** |
| --- | --- | --- | --- |
| **TITLE** | | |  |
| Title | 1 | Identify the report as a systematic review. | 1 |
| **ABSTRACT** | | |  |
| Abstract | 2 | See the PRISMA 2020 for Abstracts checklist. | 4 |
| **INTRODUCTION** | | |  |
| Rationale | 3 | Describe the rationale for the review in the context of existing knowledge. | 6-7 |
| Objectives | 4 | Provide an explicit statement of the objective(s) or question(s) the review addresses. | 6-7 |
| **METHODS** | | |  |
| Eligibility criteria | 5 | Specify the inclusion and exclusion criteria for the review and how studies were grouped for the syntheses. | 8 |
| Information sources | 6 | Specify all databases, registers, websites, organisations, reference lists and other sources searched or consulted to identify studies. Specify the date when each source was last searched or consulted. | 8 |
| Search strategy | 7 | Present the full search strategies for all databases, registers and websites, including any filters and limits used. | 8 |
| Selection process | 8 | Specify the methods used to decide whether a study met the inclusion criteria of the review, including how many reviewers screened each record and each report retrieved, whether they worked independently, and if applicable, details of automation tools used in the process. | 8 |
| Data collection process | 9 | Specify the methods used to collect data from reports, including how many reviewers collected data from each report, whether they worked independently, any processes for obtaining or confirming data from study investigators, and if applicable, details of automation tools used in the process. | 9 |
| Data items | 10a | List and define all outcomes for which data were sought. Specify whether all results that were compatible with each outcome domain in each study were sought (e.g., for all measures, time points, analyses), and if not, the methods used to decide which results to collect. | 9 |
|  | 10b | List and define all other variables for which data were sought (e.g., participant and intervention characteristics, funding sources). Describe any assumptions made about any missing or unclear information. | 9 |
| Study risk of bias assessment | 11 | Specify the methods used to assess risk of bias in the included studies, including details of the tool(s) used, how many reviewers assessed each study and whether they worked independently, and if applicable, details of automation tools used in the process. | 9 |
| Effect measures | 12 | Specify for each outcome the effect measure(s) (e.g., risk ratio, mean difference) used in the synthesis or presentation of results. | 10 |
| Synthesis methods | 13a | Describe the processes used to decide which studies were eligible for each synthesis (e.g., tabulating the study intervention characteristics and comparing against the planned groups for each synthesis (item #5)). | 10 |
|  | 13b | Describe any methods required to prepare the data for presentation or synthesis, such as handling of missing summary statistics, or data conversions. | 10 |
|  | 13c | Describe any methods used to tabulate or visually display results of individual studies and syntheses. | 10 |
|  | 13d | Describe any methods used to synthesize results and provide a rationale for the choice(s). If meta-analysis was performed, describe the model(s), method(s) to identify the presence and extent of statistical heterogeneity, and software package(s) used. | 10 |
|  | 13e | Describe any methods used to explore possible causes of heterogeneity among study results (e.g., subgroup analysis, meta-regression). | 10 |
|  | 13f | Describe any sensitivity analyses conducted to assess robustness of the synthesized results. | 10 |
| Reporting bias assessment | 14 | Describe any methods used to assess risk of bias due to missing results in a synthesis (arising from reporting biases). | 10 |
| Certainty assessment | 15 | Describe any methods used to assess certainty (or confidence) in the body of evidence for an outcome. | 10 |
| **RESULTS** | | |  |
| Study selection | 16a | Describe the results of the search and selection process, from the number of records identified in the search to the number of studies included in the review, ideally using a flow diagram. | 10-11 |
|  | 16b | Cite studies that might appear to meet the inclusion criteria, but which were excluded, and explain why they were excluded. | 10-11 |
| Study characteristics | 17 | Cite each included study and present its characteristics. | 10-11 |
| Risk of bias in studies | 18 | Present assessments of risk of bias for each included study. | 17 |
| Results of individual studies | 19 | For all outcomes, present, for each study: (a) summary statistics for each group (where appropriate) and (b) an effect estimates and its precision (e.g., confidence/credible interval), ideally using structured tables or plots. | 15-17 |
| Results of syntheses | 20a | For each synthesis, briefly summarise the characteristics and risk of bias among contributing studies. | 15-17 |
|  | 20b | Present results of all statistical syntheses conducted. If meta-analysis was done, present for each the summary estimate and its precision (e.g., confidence/credible interval) and measures of statistical heterogeneity. If comparing groups, describe the direction of the effect. | 15-17 |
|  | 20c | Present results of all investigations of possible causes of heterogeneity among study results. | 15-17 |
|  | 20d | Present results of all sensitivity analyses conducted to assess the robustness of the synthesized results. | 18 |
| Reporting biases | 21 | Present assessments of risk of bias due to missing results (arising from reporting biases) for each synthesis assessed. | 17-18 |
| Certainty of evidence | 22 | Present assessments of certainty (or confidence) in the body of evidence for each outcome assessed. | 17-18 |
| **DISCUSSION** | | |  |
| Discussion | 23a | Provide a general interpretation of the results in the context of other evidence. | 22-25 |
|  | 23b | Discuss any limitations of the evidence included in the review. | 22-25 |
|  | 23c | Discuss any limitations of the review processes used. | 26 |
|  | 23d | Discuss implications of the results for practice, policy, and future research. | 25 |
| **OTHER INFORMATION** | | |  |
| Registration and protocol | 24a | Provide registration information for the review, including register name and registration number, or state that the review was not registered. |  |
|  | 24b | Indicate where the review protocol can be accessed, or state that a protocol was not prepared. |  |
|  | 24c | Describe and explain any amendments to information provided at registration or in the protocol. |  |
| Support | 25 | Describe sources of financial or non-financial support for the review, and the role of the funders or sponsors in the review. |  |
| Competing interests | 26 | Declare any competing interests of review authors. |  |
| Availability of data, code and other materials | 27 | Report which of the following are publicly available and where they can be found: template data collection forms; data extracted from included studies; data used for all analyses; analytic code; any other materials used in the review. |  |

*From:*  Page MJ, McKenzie JE, Bossuyt PM, Boutron I, Hoffmann TC, Mulrow CD, et al. The PRISMA 2020 statement: an updated guideline for reporting systematic reviews. BMJ 2021;372: n71. doi: 10.1136/bmj. n71

For more information, visit: <http://www.prisma-statement.org>

**Supplementary Table 5: ASTAR-2 (Assessing the methodological quality of systematic reviews-2) Guidelines checklist.**


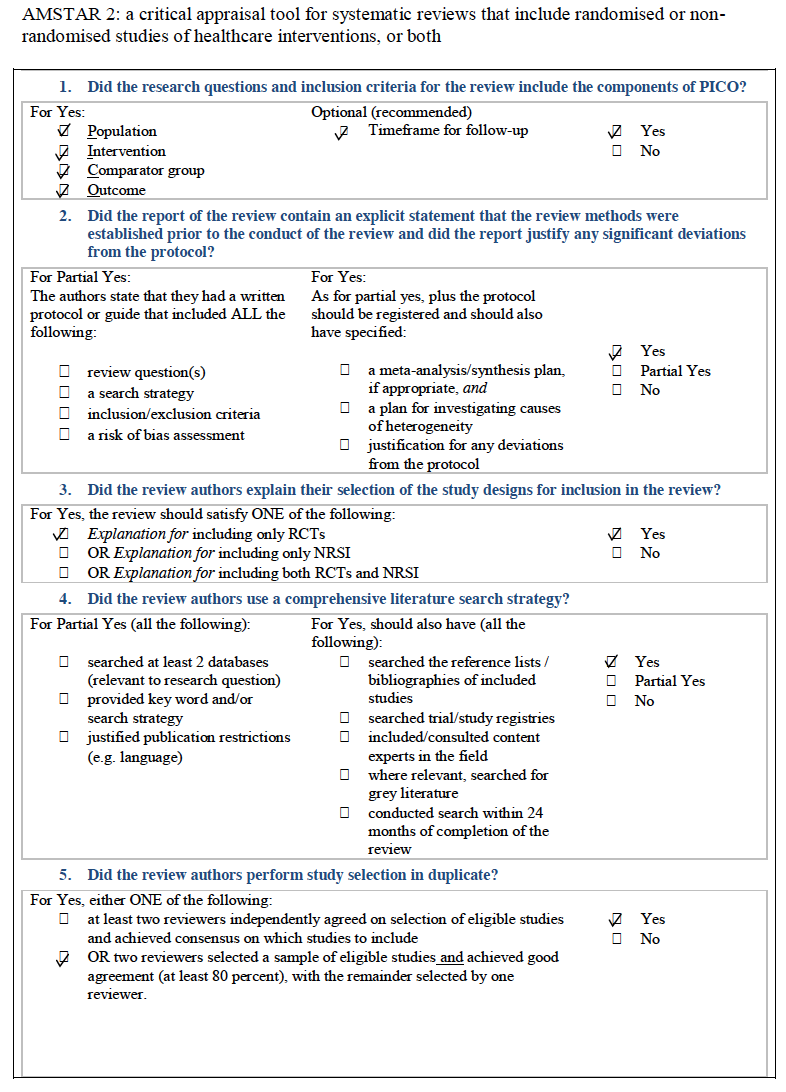


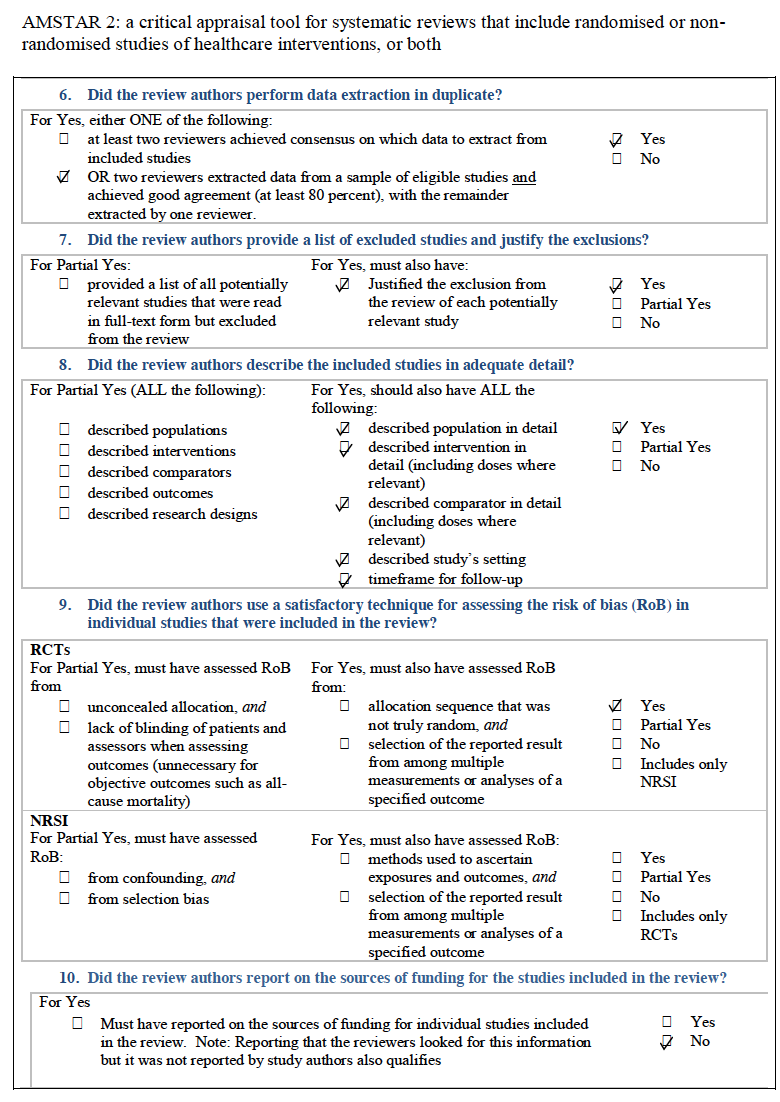


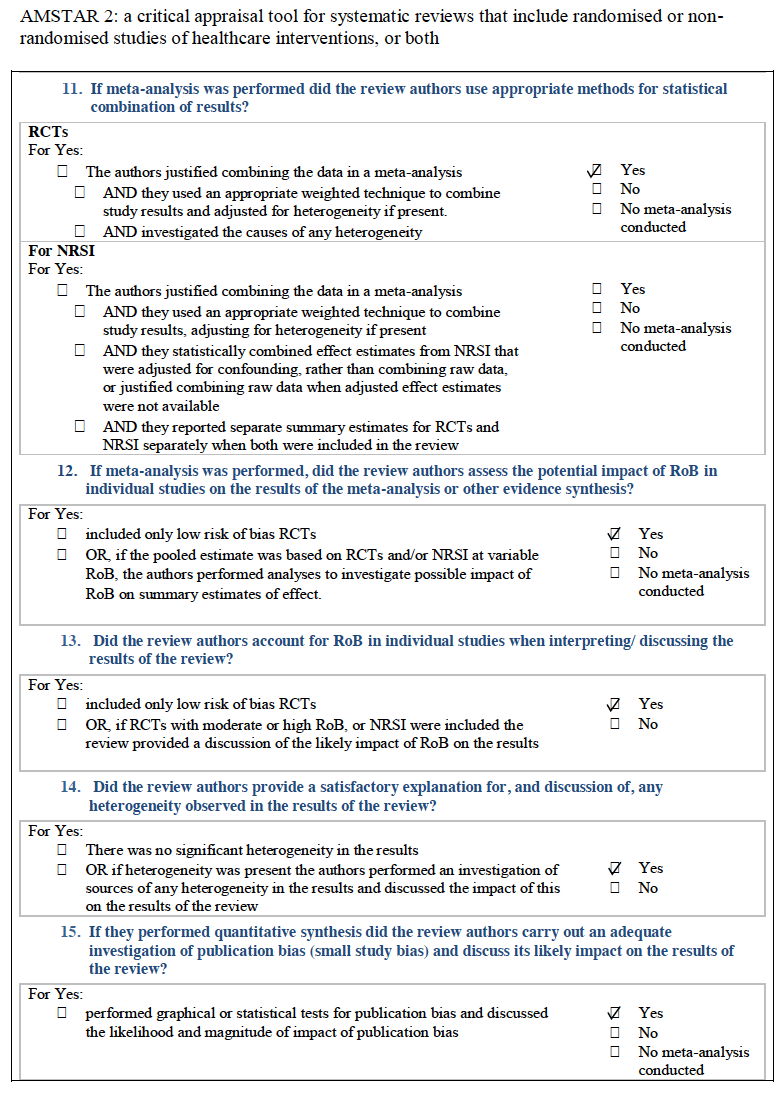


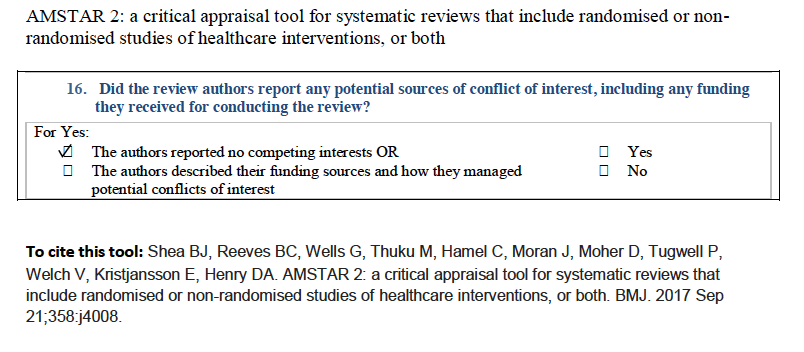

Supplement: S1 File — (DOCX) [file pone.0295804.s001.docx]
